# Investigation of absorptance and emissivity of thermal control coatings on Mg–Li alloys and OES analysis during PEO process

**DOI:** 10.1038/srep29563

**Published:** 2016-07-07

**Authors:** Zhongping Yao, Qixing Xia, Pengfei Ju, Jiankang Wang, Peibo Su, Dongqi Li, Zhaohua Jiang

**Affiliations:** 1 MIIT Key Laboratory of Critical Materials Technology for New Energy Conversion and Storage, School of Chemistry and Chemical Engineering, Harbin Institute of Technology, Harbin 150001, PR China; 2 Shanghai Aerospace Equipment Manufacture, Shanghai 200245, PR China

## Abstract

Thermal control ceramic coatings on Mg–Li alloys have been successfully prepared in silicate electrolyte system by plasma electrolytic oxidation (PEO) method. The PEO coatings are mainly composed of crystallized Mg_2_SiO_4_ and MgO, which have typical porous structure with some bulges on the surface; OES analysis shows that the plasma temperature, which is influenced by the technique parameters, determines the formation of the coatings with different crystalline phases and morphologies, combined with “quick cooling effect” by the electrolyte; and the electron concentration is constant, which is related to the electric spark breakdown, determined by the nature of the coating and the interface of coating/electrolyte. Technique parameters influence the coating thickness, roughness and surface morphology, but do not change the coating composition in the specific PEO regime, and therefore the absorptance (*α*_S_) and emissivity (*ε*) of the coatings can be adjusted by the technique parameters through changing thickness and roughness in a certain degree. The coating prepared at 10 A/dm^2^, 50 Hz, 30 min and 14 g/L Na_2_SiO_3_ has the minimum value of *α*_S_ (0.35) and the maximum value of *ε* (0.82), with the balance temperature of 320 K.

As one of the lightest structural material^1^,^2^, Magnesium-Lithium alloys show great application prospect in the fields of aeronautics and astronautics. However, the large thermal gradient between the sunlit and shadowed sides in space inflicts the performance of materials for the space instruments and equipment in orbit. So it is necessary for spacecrafts and the affiliated instruments and equipment in service to take effective thermal protection methods. Thermal control coating is usually considered as a useful passive thermal protection method, which is widely researched in recent years^3^,^4^,^5^. Because the instruments and components of in-orbit spacecraft have the different thermal control requirements due to the different work environments and the different service purposes, the controllable adjustment of absorptance and emissivity is a key factor for the design of thermal control coatings.

Plasma electrolytic oxidation (PEO) has been becoming a more and more popular technique to prepare the functional oxide coatings on the valve metals due to the excellent properties of the prepared coatings such as the controllable composition and structure, the strong adhesion strength with the substrate and the chemical stability and so on^6^,^7^,^8^. Among them, various thermal control coating on Al^9^,^10^,^11^, Mg^12^,^13^ and Ti^14^,^15^,^16^ have been successfully prepared by PEO method. For instance, Wu *et al*.^10^ prepared the thermal control coatings with low *ε/α*_s_ on Mg alloy and Al alloy, respectively and found that the *ε* of coatings were related with surface morphology. Our group^17^ obtained the high *ε/α*_s_ coatings on Ti6Al4V alloys in zirconate electrolytes and the intrinsic *ε* and *α*_s_ of two kinds of ceramic coatings were studied. However, the researches on the controllable preparation of the thermal control coatings on Mg-Li alloys is seldom reported up to now.

Besides, the PEO coating’s composition and structure and properties are really related to the electric spark breakdown mechanism under the fixed substrate alloy and the electrolyte system. In recent years, the optical emission spectroscopy (OES) has become an important tool to measure and analyze the spark discharges during the PEO process through the plasma temperature (*T*_e_) and electron concentration (*N*_e_) calculated by the feature lines of discharge ions in OES^18^,^19^,^20^,^21^,^22^,^23^. S. Stojadinović^18^ detected the OES during the PEO process on the surface of Ta alloy, and the plasma sparks were divided into anodic luminescence and strong ionization discharge. Hussein, R^22^ calculated the *T*_e_ of the sparks discharge during the PEO process of Ti alloys by the intensity of the Al lines positioned at 396.2 nm/309.1 nm, as well as the *N*_e_ by the H_β_ lines. J. Jovović^21^,^24^ studied the PEO spectra of magnesium- and aluminum- alloys, and the functions of the ions from electrolytes and metal substrate for the electric breakdown of the PEO process were discussed. However, the relation of spark discharges to the structure and performances of PEO coating have rarely been studied.

Based on the above summary, the thermal control ceramic coatings on Mg–Li alloys were successfully prepared in silicate system in this work, and the composition and structure of the coatings were characterized and the influences of the technique parameters on the thermal control properties of the coatings were investigated. Besides, the plasma spectrum characteristics was also analyzed base on the OES technique to disclose the effects of the plasma electric discharge on the structure and composition of the coatings and their relations to the adjustment of the thermal control properties.

## Experimental details

### Preparation of ceramic coatings

Plate specimens of 40 mm × 40 mm × 2 mm with a nominal composition of 4% lithium and 96% magnesium in mass fraction were mechanically polished with 240, 600, 1000 and 2000 grit SiC sandpaper, respectively, and cleaned with distilled water before the PEO treatment. A homemade single pulse power of 10 kW was used for the plasma electrolytic oxidation of the specimens in a silicate-based electrolyte under different electric parameters for different times. The plate specimens were served as an anode in a water-cooled electrochemical bath made of stainless steel which was used as a cathode. The coatings were prepared at different electric parameter, PEO reaction time and different concentrations of Na_2_SiO_3_ for different time. The duty ratio of the pulses was fixed at 20%. The temperature of the electrolyte was controlled to be below 30 °C with a cooling water flow. After PEO treatment, the obtained coatings were cleaned with distilled water and dried in air.

### Analysis of phase composition and structure

The thickness and roughness of PEO coating were investigated using thickness gauge (CTG-10, Time Company, China) and roughness tester (TR200, Time Group Inc., China). The morphology and element distribution of the coatings were analyzed by field emission scanning electron microscope (FESEM, JSM-6480, Jeol, Japan) equipped with energy dispersive X-ray spectrometer (EDS, Oxford Model 7537, America). D/Max-2400 X-ray diffraction (XRD, TTR-III, Rigaku, Japan) was used to investigate the crystallized phase of the coatings.

### The Optical emission spectroscopy

The Ocean Optics Sensor (QE6500, Ocean optics, US) was used to measure the spectrum within the wavelength range 200–900 nm during the PEO process. The total spectrum was collected with integral time 2 s to 10 s. *T*_e_ and *N*_e_ of the PEO process were calculated by the equations shown in Eqs (1a) and (2)^18^,^23^, where *I* (1) and *I* (2) are relative line intensities of the same species in question, *A*_*mn*_*(i)* the transition probabilities*, m* the upper level of the respective lines, *g*_*m*_*(i)* the statistical weight of the upper levels, *λ*_0_*(i)* the wavelengths of the line centers in vacuum, *E*_*m*_*(i)* are energies of the upper levels of lines and *k*_*B*_*T* the thermal energy^25^, *C* (*N*_e_, *T*) the extensive tables of the broadening parameters, Δλ_1/2_ the full width at half maximum (FWHM).


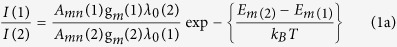



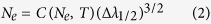


### Evaluation of thermal control property

The emissivity of the coatings was measured by a TEMP 2000, a portable infrared emissivity spectrometer in the range 250–2500 nm at room temperature. The Perkin Elmer Lambda 950 UV-Vis-NIR spectrophotometer was used to measure the absorbance of PEO coatings. The balance temperature was calculated based on Eq. (3) 16.


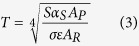


where, S is the solar constant (0.135 W/cm^2^), α_S_ the solar absorbance of the exterior surface, *A*_p_ the projected surface area in cm^2^ perpendicular to the solar rays, σ the Stefan–Boltzmann constant, *ε* (T) the emissivity, and *A*_R_ the total “effective” area in cm^2^ for heat radiation.

## Results and Discussion

### Thickness and Roughness of the coatings

**Figure 1:**
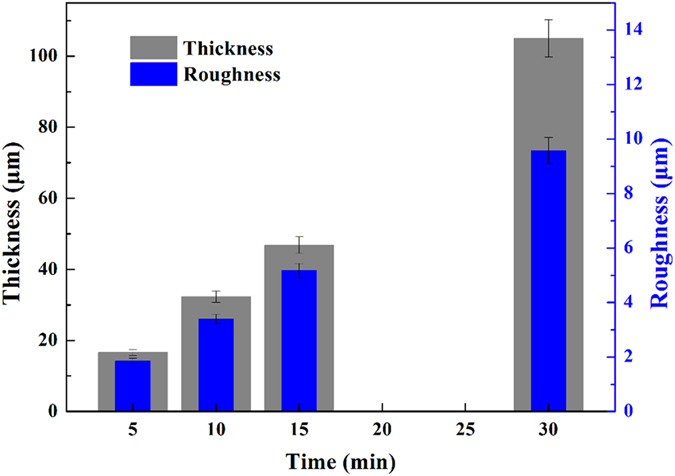
Thickness and roughness of the PEO coatings prepared under different reaction time. Current density: 10 A/dm^2^; working frequency of 500 Hz; duty ratio of 20%; electrolyte components: 10 g/L Na_2_SiO_3_·9H_2_O, 1.0 g/L NaOH and 1 g/L NaF.

The prepared coatings are milky white and uniform with the thickness from tens of microns to over 100 microns in our experiments. The roughness changes from a few microns to nearly over ten microns. Because the average roughness of the polished Mg-Li alloy samples is around 0.278 μm, which is negligible compared with that of the coatings, the coating thickness and roughness are therefore greatly influenced by the technique parameters. Generally, enhancing the electric parameters like current density, cell voltage and frequency means the more energy applied to the electrode samples, which leads to the quicker growth rate of the coatings. Increasing the electrolyte concentrations improves the solution conductivity and is liable for the migration, adsorption and sintering deposition of the ions from the electrolyte on the electrode surface. Both of them are positive for the increase of the coating thickness and roughness (please, see supplementary Table S1). Besides, the influence of the reaction time on the thickness and roughness of the coatings is shown in Fig. 1. It can be found that the thickness and roughness of the coatings is approximately linearly increased as the reaction time is increased.

### Structure and composition of the coatings

**Figure 2:**
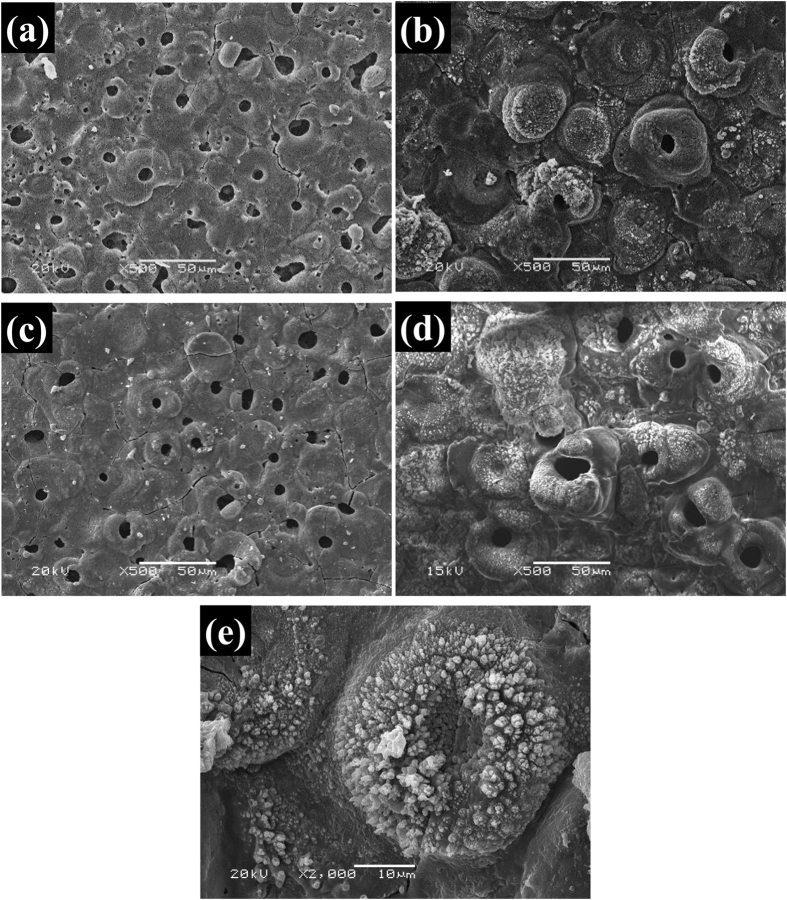
Surface SEM images of PEO coatings prepared with the working frequency of 500 Hz, the duty ratio of 20%, and electrolyte components: 10 g/L Na_2_SiO_3_·9H_2_O, 1.0 g/L NaOH and 1g/L NaF. (**a**) *i* = 10 A/dm^−2^, *t* = 5 min; (**b**) *i* = 10 A/dm^−2^, *t* = 15 min; (**c**) *i* = 5 A/dm^−2^, *t* = 30 min; (**d**) *i* = 10 A/dm^−2^, *t* = 30 min; (**e**) the enlarged picture of (**d**).

The technique parameters also influence the structure and composition of the coatings. Figure 2 shows the micro structure of the PEO coatings prepared under different current densities and reaction times, respectively. All the coatings have the porous structure. The average size of the pores, which could be observed significantly, increases from 8.1 μm to 11.8 μm with the increase of the reaction time and from 6.8 μm to 11.8 μm with the increase of the current density which was achieved through pore size statistics by ImageJ software. Apart from these comparatively large pores, a large number of small pores (in nanoscale size) are also distributing on the whole coating surface. Furthermore, there are some micro-cracks formed on the surface which may be due to the internal stress during the PEO process. Furthermore, some bulges are distributing randomly throughout the coating surface. Increasing the current density or the reaction time, the plasma discharging process is gradually enhanced and consequently much more molten coating-forming materials are ejected through the breakdown-channels and parts of pores are filled up, and therefore, the amount of micro-pores is reduced whereas the some bulges are generated, which leads to the increase of the surface roughness, which is consistent with the roughness changes of the coatings. Figure 2(e) is the partial enlarged picture of Fig. 2(d). Interestingly, there are a large number of micro/nano-sized particles gathered on the bulges, which means that the real surface area of the coatings are greatly enlarged, accompanying the increase of the roughness. Electrolyte concentration and other technique parameters present the similar effects on the micro structure of the coatings.

The surface EDS analysis of the coatings (please, see Supplementary Table S2) shows that the coatings are composed of a large amount of O, Si, Mg and a few F and Na. Obviously, O, Si, Na and F come from the electrolytic solution and Mg is from the substrate. With the increase of the current density or the reaction time, the contents of Si and Na are increased while the content of Mg is decreased, and the contents of O and F change less. Moreover, the elemental composition of the bulges shown in Fig. 2(e) is also shown in Table 1. Compared with the other data in Table 1, the bulges have much more Si and Na contents and less Mg content, which means that the formation of micro/nano-sized particles of the bulges is mainly due to the deposition of the electrolyte components.

**Figure 3:**
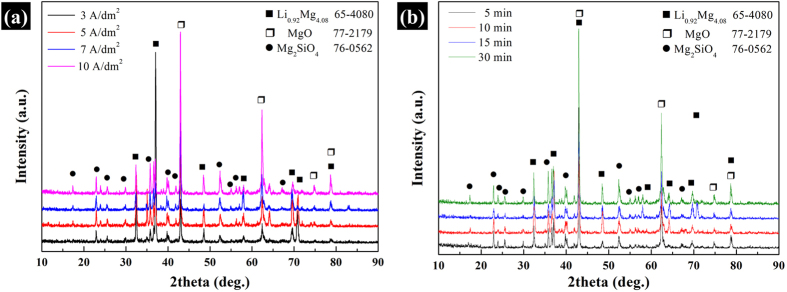
XRD patterns of the PEO coatings prepared with the working frequency of 500 Hz, the duty ratio of 20% and electrolyte components: 10 g/L Na_2_SiO_3_·9 H_2_O, 1.0 g/L NaOH and 1 g/L NaF. (**a**) current density; (**b**) reaction time.

Figure 3 shows XRD patterns of the PEO coatings prepared under different current densities and different reaction times. The diffraction peaks observed in patterns are corresponding to Li_0.92_Mg_4.08_, MgO and Mg_2_SiO_4_, respectively, so it can be confirmed that the crystallized phase of PEO coatings are MgO and Mg_2_SiO_4_ and the substrate is Li_0.92_Mg_4.08_. As the current density or the reaction time increases, the peaks of MgO and Mg_2_SiO_4_ become stronger while those of Li_0.92_Mg_4.08_ are gradually decreased due to the increase of the coating thickness. There is no crystallized substance containing Li generated in the coating. Besides, no peaks corresponding to sodium salts or silicates emerge in the patterns, and consequently they maybe exist in the form of amorphous phases.

### OES analysis during PEO process

The typical optical emission spectrum (please, see Supplementary Fig. S1 and Table S3) shows the main spectral lines of Na, Li, Mg, H and O observed in the spectroscopy, which indicates that the plasma discharges are mainly due to the elements (Na, H, O) from the solution and the substrate elements (Mg, Li). The notation I signify a neutral atom, while the notation II means the singly ionized atom. The Na 589.0 nm lines were the strongest among all of lines, corresponding to the yellow color of the discharging sparks during the PEO process.

**Figure 4:**
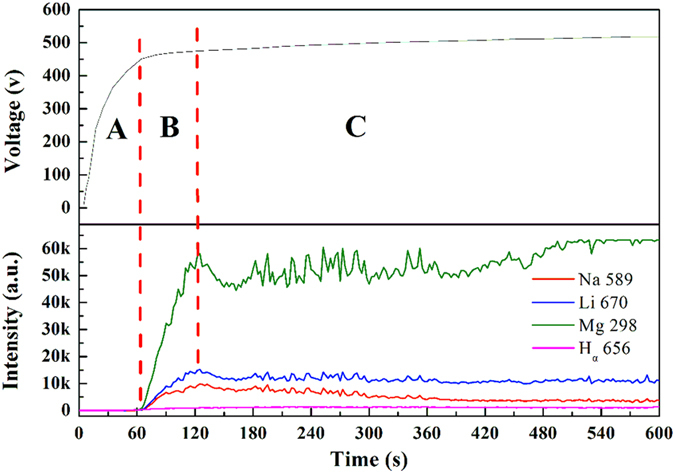
Time variations of cell voltage and intensity of Na 589, Li 670, Mg 278 and H_α_ 656 lines (Integration time 2 s).

Figure 4 is the time variations of cell voltage and the intensities of the four main peaks (Na 589, Li 610, Mg 286, H 656) during the PEO process at 10 A/dm^2^. According to the voltages curves and spectral characteristics, PEO process is divided into three stages. Stage A contains the initial 60 seconds. In this stage, the cell voltage is increased rapidly and after a very short time the only tiny and fluorescent sparks on the samples are observed by our eyes. Because of the limitation of the OES detector, the spark discharge is nearly not detected.

At the following stage B, the number of the sparks starts to increase significantly and the spark color quickly turns into yellow. Meanwhile, the PEO voltage increases a little more slowly than that at stage A. Then, the following long and stable period is named as stage C (we usually called micro arc period). At this stage both the voltage increases stably and gradually and the sparks are increased in number. Among the four spectrum lines, the Na ions are excited much more than the other ions and the Na sparks become the most distinct with the increase of the process time, which means that the electric breakdown of the PEO process is maintained mainly because of the Na ion discharges.

**Figure 5:**
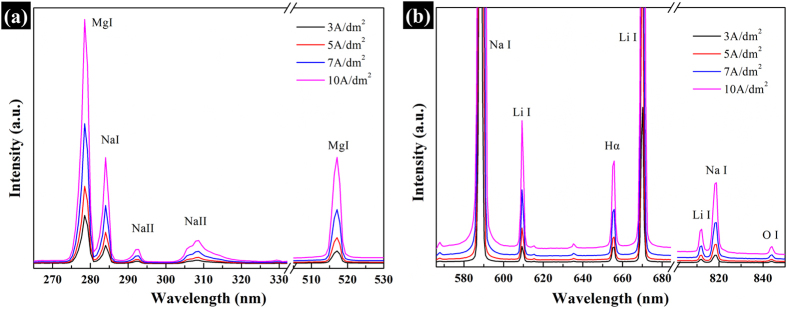
Optical emission spectrum obtained during PEO reaction at 3, 5, 7, 10 A/dm^2^ for 10 min (**a**) 270 nm–530 nm; (**b**) 580 nm–900 nm) (Integration time 10 s).

Figure 5 shows the OES patterns of the main elements detected in the process at different current densities. As the current density increases, more input energy from the electric source is applied to the specimen surface and therefore the plasma discharge is enhanced and the feature lines are correspondingly strengthened.

**Figure 6:**
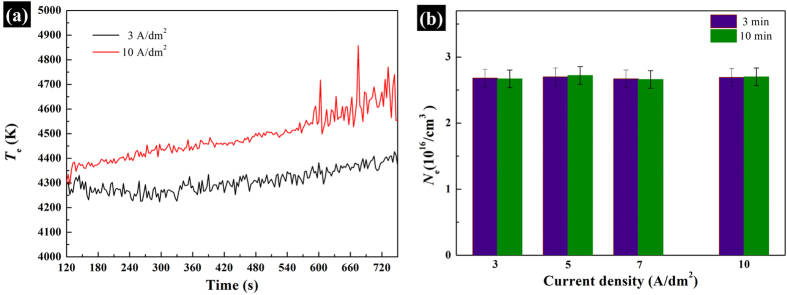
*T*_e_ (**a**) and *N*_e_ (**b**) calculated under different PEO conditions.

According to Eqs (1) and (2), the plasma temperatures (*T*_e_) and the electron concentrations (*N*e) were calculated based on Na ions peaks and H_β_ peak, respectively, with the results of *T*_e_ and *N*_e_ shown in Fig. 6. It can be noted that the value of *T*_e_ is about between 4000 K and 5000 K, which increases gradually over time. Besides, the value of *T*_e_ is improved about 150 K–300 K by increasing current density from 3 A/dm^2^ to 10 A/dm^2^. However, *N*_e_ remains stable with the average value of 2.68 × 10^16^/cm^3^, no matter how current density or reaction time changes.

During the plasma discharging process, the plasma temperatures determines the heat effect in the discharging micro areas, which influences the composition, structure and surface morphologies. The melting points of MgO and Mg_2_SiO_4_ are 3073 K and 2773 K, respectively, which are much lower than the plasma temperature (*T*_e_), therefore theses high-temperature stable crystal phases are generated in the coating. In addition, it is well known that Mg_2_SiO_4_ and MgSiO_3_ could be regarded as the combination of MgO and SiO_2_. Under such high plasma temperature in the micro discharging zone, salt silicate can be decomposed into SiO_2._ The formation of MgO in the coating indicates that MgO is comparatively adequate due to the oxidation of the substrate. So, much more melting MgO could combine with SiO_2_ to form Mg_2_SiO_4_ instead of MgSiO_3_, accordingly the following equation (1b).


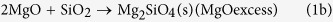


That is why the crystallized MgSiO_3_ with the melting point of 1830 K is not generated under such extreme conditions. On the other hand, the amorphous substance containing elements like Na and F are also formed through the quick-cooling effect of the electrolyte and this may be due to the cooling rate of the electrolyte higher than the nucleation rate of these substances. Furthermore, Li_0.92_Mg_4.08_ corresponding to the substrate shows that the micro-area plasma discharging process does not influence the structure and composition of the substrate, which just is one of various advantage of PEO techniques.

Besides, the formation of the bulges on the coating surface and the increase of the coating thickness and roughness are also attributed to the enhancement of the heat effects by the gradual increase of the plasma temperature with the reaction time and current density and the quick-cooling effect of the electrolyte as well. The repeated melting, sintering and cooling solidifying of the micro reaction zones on the coating during the PEO process do much contribution on the formation of the roughness surface and the pores and the increase of the coating thickness as well.

Generally, the electron concentrations (*N*e) is determined by the natures of PEO regime under the fixed substrate/coating/electrolyte interface and the specific electrolyte system. Once the avalanche electronic current reaches a certain critical value, the electric breakdown occurs. Therefore, *N*_e_ is corresponding to the breakdown current density (*i*_break_), which approximatively represents the avalanche electronic current, reflecting this critical value. So, it is only mainly responsible for the electric breakdown of PEO process and does less contribution on the coating growth and structure. On the other hand, the total current density (*i*) during PEO process can be simply divided into two parts, *i.e. i* = *i*_break_ + *i*_growth_. The *i*_break_ is used for the electric breakthrough of the coatings (discussed above) while *i*_growth_ is for the growth of coatings. *i*_growth_ is related to the migration rate of the ions in the electrolyte system, the anodic oxidation dissolution rate from the substrate and the deposition rate of all the ions above. The proper enhancement of the electric parameters or the extending of the reaction time, which means that although the increase of the applied energy by the power source, does not change *i*_break_, but it is helpful for the increase of *i*_growth_ and the improvement of the plasma temperature (*T*_e_) of the micro reaction zones, which consequently influences the growth rate, the crystallization and phase transformation and the structure and morphology of the coatings.

### Thermal control properties of the coatings

#### Effects of technique parameters on absorptance and emissivity

In general, the thermal control properties of the coatings are mainly determined by emissivity (ε) and solar absorbance (*α*_s_). Different thermal control requirements of the specific instrument or equipment can be fulfilled by the adjustment of absorptance and emissivity. Table 1 shows the effects of the technique parameters on absorptance and emissivity of the coatings. Clearly, increasing the working frequency, the emissivity increases first and then decrease while the absorptance presents a slight declining trend. Secondly, increasing the current density, the reaction time or the concentration of NaSiO_3_.9 H_2_O, the emissivity increases while the absorptance decreases slowly. Moreover, when the reaction time is 15–30 min, or the concentration of NaSiO_3_.9 H_2_O is 14–20 g/L, the emissivity of the coatings is basically similar due to the controllable limitation of of these technique parameter.

Generally, the thermal control performance is related to the composition and structure of the coatings. For a given PEO regime in this experiment, the composition for all the coatings is similar according to EDS and XRD analysis. Therefore, the thermal control properties are mainly determined by their structure and morphology, especially the macro-sized structure like the thickness and the roughness^15^,^16^,^17^,^26^. The increase of the thickness and roughness means the increase of the real radiation surface area of the porous coatings, consequently improving the emissivity of the coatings. On the other hand, the size of the pores are between a few microns and dozens of micrometers which are much larger than the wavelength measurement range of the solar absorption wave. This illustrates that when the light goes into these pores, the mirror reflection happens and therefore these pores have no contribution on the absorption of light. What actually influences the light absorption are the cracks and the bulges within the micro/nano - sized particles on the coating surface. When the light goes into the cracks or the gaps formed by these micro/nano - sized particles, diffuse reflection and refraction of the light will happen many times to extend the staying time which are liable for the absorption by the coatings.

#### Balance Temperature of the thermal control coatings

**Figure 7:**
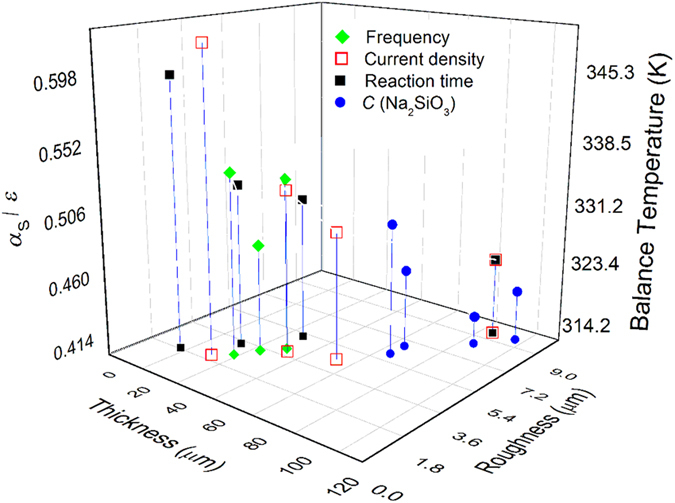
Dependency of αs/ε and the balanced temperature (K) on thickness and roughness of the coatings.

Balanced temperature is another important factor to evaluate the thermal control properties of the coatings, which is calculated based on the ratio of absorptance and emissivity (*α*_s_/ε) according to Eq. (3). Figure 7 is the dependency of *α*_s_/ε and the balanced temperature (K) on thickness and roughness of the coatings. Obviously, balance temperature is strongly related to the thickness and roughness of the coatings, which decrease with the increase of the thickness and roughness. The balance temperature can be adjusted in the range of around 320 K–350 K through PEO technique parameters to change the thickness and roughness of the coatings. Besides, current density and reaction time presents the larger adjustment ability than concentration of NaSiO_3_.9H_2_O and working frequency.

## Conclusions

1. The PEO coatings on Mg-Li alloy have typical porous structure with some bulges on the surface, with the main crystalline phase of Mg_2_SiO_4_ and MgO. With the enhancement of the electric parameters, the increase of reaction time and the concentration of Na_2_SiO_3_, the thickness and roughness is increased. The bulges have numerous micro/nano-sized particles containing higher content of Na and Si than the other part of the coatings.
2. OES analysis shows that plasma temperatures (*T*_e_) is about between 4000 K and 5000 K and the electron concentrations (*N*_e_) is 2.68 × 10^16^/cm^3^. The former is slightly influenced by the electric parameters and reaction time, which explains the crystallized and amorphous composition and structure and surface morphology of the coatings, combined with the quick-cooling effect of the electrolyte. The latter is determined by the natures of PEO regime and mainly used for the electric discharge.
3. The thermal control properties of the coatings are strongly dependent on the thickness and roughness of the coatings, which can be adjusted by the technique parameters. The balance temperature of the coatings is in the range of around 320 K–350 K, which gradually decreases with the increase of the thickness and roughness. Current density and reaction time show a better adjustment ability for the thermal control properties of the coatings than other technique parameters.

## Additional Information

**How to cite this article**: Yao, Z. *et al*. Investigation of absorptance and emissivity of thermal control coatings on Mg–Li alloys and OES analysis during PEO process. *Sci. Rep.* **6**, 29563; doi: 10.1038/srep29563 (2016).

## Supplementary Material

Supplementary Information

## Figures and Tables

**Table 1:** Absorbance and emissivity of the coatings prepared under different technique parameters.

Technique parameter		ε	*α*_s_
Frequency* (Hz)	50	0.81	0.43
	500	0.85	0.41
	1000	0.77	0.40
Current density** (A.dm^−2^)	3	0.67	0.42
	5	0.72	0.37
	7	0.76	0.37
	10	0.80	0.36
	5	0.71	0.42
Reaction time*** (min)	10	0.77	0.40
	15	0.80	0.40
	30	0.80	0.37
	7	0.78	0.39
C (Na_2_SiO_3_)**** g/L	10	0.80	0.37
	14	0.82	0.35
	20	0.82	0.36

^*^Duty ratio: 20%; Working frequency is 50Hz; Reaction time: 30 min; Electrolyte components: NaOH 1 g/L, NaSiO_3_.9H_2_O 10 g/L and NaF 1 g.

^**^Duty ratio 20%; Current density 10 A.dm^−2^; Reaction time: 30 min; Electrolyte components: NaOH 1 g/L, NaSiO_3_.9H_2_O 10 g/L and NaF 1 g.

^***^Duty ratio 20%; Current density 10 A.dm^−2^; Current density 10 A.dm^−2^; Electrolyte components: NaOH 1 g/L, NaSiO_3_.9 H_2_O 10 g/L and NaF 1 g.

^****^Duty ratio 20%; Working frequency is 50Hz; Current density 10 A·dm^−2^; Reaction time: 30min; Other electrolyte components: NaOH 1 g/L and NaF 1 g.
