# Supplementary material for: Investigation of absorptance and emissivity of thermal control coatings on Mg–Li alloys and OES analysis during PEO process
Source: Sci Rep. 2016 Jul 7;6:29563. doi: 10.1038/srep29563 (PMC4935892; doi:10.1038/srep29563)
Supplement: Supplementary Information [file srep29563-s1.doc]

**Supporting Information**

for

“Investigation of absorptance and emissivity of thermal control coatings on Mg–Li alloys and OES analysis during PEO process”

Zhongping Yao^1^,[[1]](#footnote-2) , Qixing Xia^1^, Pengfei Jiu^2^, Jiankang Wang^1^, Peibo Su^2^, Dongqi Li^1^ and Zhaohua Jiang^1^

^1^School of Chemical Engineering and Technology, Harbin Institute of Technology, Harbin 150001, PR China.

^2^Shanghai Aerospace Equipment Manufacture, Shanghai 200245, PR China

**SI Table 1** Thickness and roughness of the coatings prepared under different technique parameters

|  |  | Thickness (μm) | Ra (μm) |
| --- | --- | --- | --- |
| Working frequency  (Hz) | 50 | 38.3 | 2.62 |
|  | 500 | 43.5 | 3.41 |
|  | 1000 | 50.1 | 4.08 |
| Current density  (A/dm^2^) | 0.3 | 39.7 | 2.009 |
|  | 0.5 | 55.7 | 3.549 |
|  | 0.7 | 72.2 | 4.479 |
|  | 1.0 | 70.5 | 5.324 |
| Reaction time  (min) | 5 | 16.6 | 1.86 |
|  | 10 | 32.3 | 3.40 |
|  | 15 | 46.8 | 5.17 |
|  | 30 | 105 | 9.58 |
| C(Na_2_SiO_3_)  (g/L) | 7 | 85.1 | 5.954 |
|  | 10 | 85.0 | 6.754 |
|  | 14 | 105.0 | 8.406 |
|  | 20 | 116.0 | 9.580 |

**SI Fig. 1** Optical emission spectrum obtained during PEO reaction of Mg-Li alloys at 10 A/dm^2^, 10 min (Integration time 2 s)

**SI Table 2** Distribution characteristics of elements on the surface of PEO coatings (at. %)

| Sample | O | F | Na | Mg | Si |
| --- | --- | --- | --- | --- | --- |
| *i* = 3 A/dm^-2^, *t* = 30 min | 56.89 | 4.17 | 1.59 | 30.38 | 6.97 |
| *i* = 5 A/dm^-2^, *t* = 30 min | 53.60 | 5.24 | 1.93 | 31.41 | 7.82 |
| *i* = 7 A/dm^-2^, *t* = 30 min | 55.17 | 4.69 | 2.88 | 28.95 | 8.32 |
| *i* = 10 A/dm^-2^, *t* = 30 min | 55.71 | 3.60 | 3.56 | 27.81 | 9.31 |
| *i* = 10 A/dm^-2^, *t* = 5 min | 56.81 | 2.16 | 1.58 | 30.84 | 8.61 |
| *i* = 10 A/dm^-2^, *t* = 10 min | 55.25 | 2.94 | 1.96 | 32.42 | 7.43 |
| *i* = 10 A/dm^-2^, *t* = 15 min | 53.36 | 4.82 | 3.47 | 29.86 | 8.49 |
| Micro/nano-particles | 55.95 | 4.92 | 8.93 | 13.65 | 16.56 |

SI Table 3 The peaks emerged in PEO spectroscopy of SI Fig. 5 (a) with the wavelength (λ), the transitions probabilities (*A*_ki_), statistical weights of the upper states *g*_k_

| Line | Λ, nm | *A*_ki_ (10^8^S^-1^) | Transition | g_k_ | Energy (eV) |
| --- | --- | --- | --- | --- | --- |
| NaI | 285.3 | 0.00554 | 5*p* ^2^P→3*s* ^2^S | 4 | 4.345 |
| NaI | 589.0 | 0.614 | 3*p* ^2^P→3*s* ^2^S | 4 | 2.104 |
| NaI | 819.5 | 0.54 | 3*d* ^2^D→3*p* ^2^P | 6 | 3.617 |
| NaII | 309.3 | - | 2*p*^5^3*p* ^3^D→2*p*^5^3*s* ^3^P | 7 | 4.008 |
| LiI | 610.4 | 0.686 | 3*d* ^2^D→2*p* ^2^P | 6 | 2.031 |
| LiI | 670.8 | 0.369 | 2*p* ^2^P→2*s* ^2^S | 2 | 1.848 |
| LiI | 812.6 | 0.349 | 3*s* ^2^S→2*p* ^2^P | 2 | 1.525 |
| MgI | 278.0 | 3.92 | 3*p*2 ^3^P→3*s*3*p* ^3^P | 5 | 4.459 |
| MgI | 383.3 | 1.68 | 3*s*3*d* ^3^D→3*s*3*p* ^3^P | 7 | 3.229 |
| MgI | 517.3 | 0.346 | 3*s*4*s* ^3^S→3*s*3*p* ^3^P | 3 | 2.396 |
| MgII | 448.1 | 2.23 | 4*f* ^2^F→3*d* ^2^D | 8 | 2.766 |
| H_β_ | 486.1 | 0.1719 | 4*d* ^2^D→2*p* 2P | 4 | 2.550 |
| H_α_ | 656.3 | 0.6465 | 3*d* ^2^D→2*p* ^2^P | 6 | 1.889 |
| OI | 777.2 | 0.369 | 2*s*^2^2*p*^3^(^4^S)3*p* ^5^P →2*s*^2^2*p*^3^(^4^S)3*s* ^5^S | 7 | 1.594 |
| OI | 844.6 | 0.322 | 2*s*^2^2*p*^3^(^4^S)^3^*p* ^3^P →2*s*^2^2*p*^3^(^4^S)3*s* ^3^S | 1 | 1.468 |

1. Corresponding author, email: yaozhongping@hit.edu.cn [↑](#footnote-ref-2)
